# Protocol for the process evaluation of a complex intervention designed to increase the use of research in health policy and program organisations (the SPIRIT study)

**DOI:** 10.1186/s13012-014-0113-0

**Published:** 2014-09-27

**Authors:** Abby Haynes, Sue Brennan, Stacy Carter, Denise O’Connor, Carmen Huckel Schneider, Tari Turner, Gisselle Gallego, the CIPHER team

**Affiliations:** Sax Institute, 235 Jones Street, Ultimo, NSW 2007 Australia; Australasian Cochrane Centre, School of Public Health and Preventive Medicine, Monash University, The Alfred Centre, 99 Commercial Road, Melbourne, VIC 3004 Australia; Centre for Values, Ethics and the Law in Medicine (VELiM), Medical Foundation Building, Sydney School of Public Health, University of Sydney, 92-94 Parramatta Road, Camperdown, NSW 2006 Australia; Menzies Centre for Health Policy, Victor Coppleson Building, University of Sydney, Camperdown, NSW 2006 Australia; World Vision Australia, 1 Vision Drive, Burwood East, VIC 3151 Australia; Centre for Health Research, School of Medicine, Building 3, Campbelltown Campus, University of Western Sydney, Penrith, NSW 2751 Australia; Faculty of Health Science, University of Sydney, 75 East Street, Lidcombe, NSW 2141 Australia

**Keywords:** Process evaluation, Complex intervention, Implementation, Knowledge exchange, Health policy, Organisational change, Capacity building, Qualitative methods, Developmental evaluation, Framework analysis

## Abstract

**Background:**

Process evaluation is vital for understanding how interventions function in different settings, including if and why they have different effects or do not work at all. This is particularly important in trials of complex interventions in ‘real world’ organisational settings where causality is difficult to determine. Complexity presents challenges for process evaluation, and process evaluations that tackle complexity are rarely reported. This paper presents the detailed protocol for a process evaluation embedded in a randomised trial of a complex intervention known as SPIRIT (Supporting Policy In health with Research: an Intervention Trial). SPIRIT aims to build capacity for using research in health policy and program agencies.

**Methods:**

We describe the flexible and pragmatic methods used for capturing, managing and analysing data across three domains: (a) the intervention as it was implemented; (b) how people participated in and responded to the intervention; and (c) the contextual characteristics that mediated this relationship and may influence outcomes. Qualitative and quantitative data collection methods include purposively sampled semi-structured interviews at two time points, direct observation and coding of intervention activities, and participant feedback forms. We provide examples of the data collection and data management tools developed.

**Discussion:**

This protocol provides a worked example of how to embed process evaluation in the design and evaluation of a complex intervention trial. It tackles complexity in the intervention and its implementation settings. To our knowledge, it is the only detailed example of the methods for a process evaluation of an intervention conducted as part of a randomised trial in policy organisations. We identify strengths and weaknesses, and discuss how the methods are functioning during early implementation. Using ‘insider’ consultation to develop methods is enabling us to optimise data collection while minimising discomfort and burden for participants. Embedding the process evaluation within the trial design is facilitating access to data, but may impair participants’ willingness to talk openly in interviews. While it is challenging to evaluate the process of conducting a randomised trial of a complex intervention, our experience so far suggests that it is feasible and can add considerably to the knowledge generated.

**Electronic supplementary material:**

The online version of this article (doi:10.1186/s13012-014-0113-0) contains supplementary material, which is available to authorized users.

## Background

There is global interest in ensuring that health policy and program development is informed by reliable research [1]. Previous studies have improved our understanding of the constraints that policymakers and program developers face in their efforts to use research [2–4], and tools have been developed to support these efforts [5], but there is still little evidence about what strategies are most effective in building individual or organisational capacity to use research more effectively [6,7]. Even less is known about how and why such strategies work and what makes them effective in one context but not another [8]. SPIRIT (Supporting Policy In health with Research: an Intervention Trial) was developed to address this pressing need (see Additional file 1 for a glossary of terms used in this article) [9].

### Supporting Policy In health with Research: an Intervention Trial (SPIRIT)

SPIRIT is testing the effects of a year-long multi-component intervention designed to increase the capacity of health policy agencies to use research. SPIRIT uses a stepped wedge cluster randomised trial design. Six government agencies that develop and implement state-wide or national health policies and programs located in Sydney, Australia, will receive the intervention. Between 15 to 60 staff are expected to participate at each site. All agencies receive six intervention components: (i) audit, feedback and goal setting; (ii) a leadership program; (iii) organisational support for research; (iv) the opportunity to test systems for accessing research and reviews; (v) research exchanges; and (vi) educational symposia for staff. The development of these components was informed by change principles, as shown in Table 1.

**Table 1:** **SPIRIT intervention components, subcomponents, and change principles**

**Intervention components**	**Subcomponents**	**Change principles underpinning each subcomponent**
**1. Audit, feedback and goal setting**	a. Feedback forum	• Engages agencies in owning and driving the program
		• Provides feedback about current practice
		• Provides a clear rationale for change
	b. Intervention selection	• Develops agreement about concrete and specific change goals
		• Is tailored to focus on the agency’s priorities
	c. Identification of other strategies	• Develops agreement about concrete and specific change goals
	d. Mid-intervention feedback	• Engages agencies in owning and driving the program
		• Monitors and provides feedback about change during the intervention
	e. SPIRIT newsletter	• Monitors and provides feedback about change during the intervention
**2. Leadership program**	a. Supporting organisational use of evidence	• Addresses systems, operations, structures and relations
		• Engages agencies in owning and driving the program
		• Develops agreement about concrete and specific change goals
	b. Leading organisational change	• Provides self-education opportunities and access to resources
		• Recognises the expertise of participants
		• Is interactive with a focus on shared reflection and problem solving
		• Uses credible, dynamic experts as presenters
**3. Organisational support for research**	a. Quarterly email endorsement from CEO	• Engages agencies in owning and driving the program
		• Uses champions to model and promote the use of evidence from research
		• Monitors and provides feedback about change during the intervention
	b. Access to WebCIPHER	• Provides self-education opportunities and access to resources
		• Uses champions to model and promote the use of evidence from research
	c. Resources for improving agency’s use of research	• Provides self-education opportunities and access to resources
		• Provides opportunity for rehearsal and practice
**4. Opportunity to test systems for accessing research and reviews (brokered services)**	a. Brokered commission of a rapid review, evaluation plan or linked data analysis	• Provides opportunity for rehearsal and practice
		• Is tailored to focus on the agency’s priorities
		• Recognises the expertise of participants
		• Provides self-education opportunities and access to resources
**5. Research exchange**	a. Interactive forum	• Is tailored to focus on the agency’s priorities
		• Recognises the expertise of participants
		• Is interactive with a focus on shared reflection and problem solving
		• Provides self-education opportunities and access to resources
		• Uses champions to model and promote the use of evidence from research
		• Uses credible, dynamic experts as presenters
	b. Summary of systematic reviews	• Is tailored to focus on the agency’s priorities
		• Provides self-education opportunities and access to resources
**6. Educational symposia for staff**	a. Valuing research symposium	• Recognises the expertise of participants
		• Is interactive with a focus on shared reflection and problem solving
		• Provides self-education opportunities and access to resources
		• Uses champions to model and promote the use of evidence from research
		• Uses credible, dynamic experts as presenters
	b. Agencies can chose two symposia from: Access to research/Appraising research/Evaluation/Working with researchers	• Recognises the expertise of participants
		• Is interactive with a focus on shared reflection and problem solving
		• Provides self-education opportunities and access to resources
		• Uses champions to model and promote the use of evidence from research
		• Uses credible, dynamic experts as presenters

The components include content that is tailored to suit the interests and needs of each agency but have standardised essential elements (i.e., the hypothesised ‘active ingredients’ of the intervention). We assume that, for the intervention to be optimally effective, the essential elements of each component should be delivered in each agency. The design of the SPIRIT intervention is based on a program logic model which outlines how the intervention is hypothesised to bring about change. Proximal and distal outcomes include:

1. Organisational capacity to use research (individual knowledge and skills; staff perceptions of the value of research; and organisational support for the use of research as demonstrated through leadership support, policies, tools and systems);
2. Research engagement actions (accessing and appraising research; generating new analyses and research including evaluation of current programs and policies; and interacting with researchers);
3. Research use (the different ways research informs policy or program work).

The outcome measures comprise an online survey and two structured interviews. A detailed description of SPIRIT, including the program logic model, is available online at http://bmjopen.bmj.com/content/4/7/e005293.full#F1 [9].

### Process evaluation of interventions to increase the use of research in health policy and program agencies

A detailed process evaluation is being conducted as part of the evaluation of SPIRIT. High quality process evaluations are critical for interpreting the outcomes of trials of complex interventions [10,11] where there is seldom a clear causal chain [12,13]. Process evaluations are increasingly used in trials of complex interventions [12,14–16], including those that seek to change professional behaviours in complex settings [17-19].

### Aims and objectives

The primary aim of the process evaluation is to describe how the SPIRIT intervention works in different settings, including if and why it has different effects or does not work at all. This will help us interpret the outcomes of the SPIRIT trial and optimise the design of future interventions. We conceptualise this work as focusing on the interaction between three Domains: 1. the intervention as it was implemented; 2. how people participated in and responded to the intervention; and 3. the contextual characteristics that mediated this relationship. Our specific objectives address each of these Domains as follows:

1. To document how the intervention was implemented, and the extent to which it was implemented as intended over time and across different intervention settings, including the degree to which essential elements were delivered (implementation fidelity). This allows us to see implementation successes or failures that may affect outcomes. (Domain 1: Implementation).
2. To describe how people participated in and responded to the intervention, including any variations across the settings [17,20]. This enables us to critique the program design and delivery, and helps with the interpretation of study outcomes. (Domain 2: Participation and response).
3. To describe the contexts in which the intervention was delivered and explore contextual factors that may influence the delivery or impact of the intervention, and the outcomes. This provides evidence about ‘real world’ feasibility and may enable findings to be transferred to other contexts [21-23]. It may also explain intentional and unintentional differences in delivery. Reflecting on the relationship between organisational context and how each agency used the program to address local needs may have implications for future program design and delivery. (Domain 3: Context).

In addition, we address a fourth objective required for new interventions in which the program theory is untested and the process evaluation is designed from scratch rather than employing piloted methods:

4. To explore how well the theory underpinning the intervention was (a) realised in the design and (b) delivered in each participating agency:
  - We will collect data that confirms, refutes or adds nuance to the constructs and relationships proposed in the SPIRIT Action Framework. This model was used as the basis for designing and testing SPIRIT intervention strategies (Redman S, Turner T, Davies HTO, Williamson A, Haynes A, Brennan S, Milat A, O’Connor D, Blyth F, Jorm L: The SPIRIT Action Framework: A structured approach to selecting and testing strategies to increase the use of research in policy, submitted).
  - We will explore whether the essential elements captured and delivered the change principles which informed them (theoretical fidelity). This includes assessing whether the elements thought to be essential appeared to be essential in real world contexts, describing how well they delivered the program’s change principles, and developing hypotheses about how amended or different essential elements might have better delivered the change principles. This information can inform future intervention development. Details of the development and testing of essential elements during this trial will be reported separately.

## Methods/Design

The process evaluation is being conducted as an integral part of the trial in each of the six participating agencies. An evaluation officer leads the work, including data collection and management. She works with a small multidisciplinary sub-team of investigators who designed the process evaluation, and who continue to monitor its implementation and contribute to the ongoing data analysis. This team is not involved in the design or implementation of the intervention.

The development of the Domains, research questions and data collection methods (including the use of interview questions, feedback form items, observation frame questions and draft indexing categories, which are discussed later) were informed by the SPIRIT study aims and change principles, and the literature on process evaluation [10–12,16–18,20–22,24–26], research utilisation [7,27–34], adult and organisational learning [35–40], and complex systems theory [14,41–43].

### Process evaluation design

We have designed a mixed methods process evaluation: gathering quantitative measures of intervention activities (such as numbers of participants and delivered components) [26], and qualitative exploration of the interaction between the intervention, how people experience it, and the contextual characteristics of the six organisations in which it is being delivered [44,45]. Table 2 provides an overview of these activities, which are discussed in more detail below.

**Table 2:** **Process evaluation domains, research questions and data collection**

**Process evaluation domains**	**Research questions**	**Core information sought**	**Data type**	**Data source**	**Record kept**
**Domain 1:** Implementation	1. How was the intervention implemented in each agency? Including:	Intervention components selected and how agencies asked that they be tailored	Updates	SPIRIT staff implementing the intervention	Fieldnotes and memos - data indexed in NVivo
	a. What components were delivered?	What was delivered in each agency, including: which components; delivery format; provider recruitment and preparation; learning materials; the fidelity with which *essential elements* were delivered; any changes to the plan, and follow-up activities	Structured observation	Intervention sessions	Completed checklists – data input in spreadsheet
	b. To what extent were the *essential elements* implemented?		Email information	SPIRIT trial coordinator’s records	Collated spreadsheet data on email delivery
			Knowledge brokering records	SPIRIT staff delivering brokered services	Brokered service assessment form
**Domain 2:** Participation and response	2. How did people interact with the intervention? What were their levels of participation and satisfaction?	Session participation and responses, including: roles of attendees, proportion of invitees who attended; nature of participation (types and extent of interaction)	Pre-session sign-in and consent process	Participants attending each session	Completed sign-in/consent sheets –no.s and roles input in spreadsheet
			Semi-structured and structured observation	Intervention sessions	Completed checklist, fieldnotes and memos – data indexed in NVivo
	3. What effects that are not captured by the outcome measures did the intervention have (including unexpected effects)?	Participants’ evaluation of intervention sessions	Self-reported evaluation feedback	Participants attending each session	Completed feedback forms – data input in spreadsheet
			Informal conversations after sessions	Liaison Person and ad hoc participants	Fieldnotes – data indexed in NVivo
		How participants and the organisational system responded to the intervention overall (including unexpected effects)	Interviews	Purposively sampled participants, Liaison Person and CEO	Audio recordings, transcripts and memos – data indexed in NVivo
			Interviews, meetings and informal conversations	SPIRIT intervention staff and providers	Fieldnotes and memos – data indexed in NVivo
**Domain 3:** Context	4. What was the context of the agencies in which the intervention was implemented?	Immediate characteristics of session delivery context (site, facilities, etc.)	Structured observation	Intervention sessions	Completed checklist fieldnotes data input in spreadsheet
		Organisational context: (i) agency culture, (ii) agenda-setting & prioritisation, (iii) leadership styles & perceptions of leaders, (iv) how research & other information is valued, accessed & used, (v) barriers and enablers to using research, (vi) other contextual factors that may affect outcomes	Semi-structured observation	Intervention sessions	Audio recordings, fieldnotes and memos – data indexed in NVivo
			Interviews	Purposively sampled participants	Audio recordings, transcripts and memos – data indexed in NVivo
			Interviews, meetings and informal conversations	SPIRIT staff implementing the intervention	Audio recordings, fieldnotes and memos – data indexed in NVivo
**Across domains**	5. How might the relationships between the program, the people and the context in each agency have shaped variations in these effects?				Analytic synthesis of above data
	6. What lessons can we derive from this study that might be relevant for other interventions and settings?				

Our approach incorporates aspects of developmental evaluation [46,47]. Traditional process evaluation tends to align with program logic models and focus largely on documenting key aspects of these linear and predictive pathways. Developmental evaluation takes a more emergent perspective, assuming that implementation within complex organisational systems will be unpredictable and will result in local adaptation, which may be more appropriate for achieving the intended program goals in that context. This approach focuses on reflective learning at every stage of the evaluation, adapting evaluation questions and data collection methods as the program is implemented, and feeding them back into an evolving trial design. This is appropriate when trialling complex strategies that are untested, producing uncertainty about what will work, where, and with whom; and when new questions, challenges, and opportunities are likely to surface [46,47]. Core aspects of SPIRIT are intended to be standardised, so process evaluation data will not be fed back to the intervention implementation team during the trial. Nevertheless, we hypothesise SPIRIT staff and providers are likely to adapt (intentionally and unintentionally) as they interact with participants and respond to contextual opportunities and constraints. The developmental evaluation perspective helps us see this variation as more than non-adherence to the implementation plan: it is expected emergence. Thus we are exploring why expert providers make particular in-situ changes, and striving to learn from how different strategies play out. These data are collated and analysed to support post-trial critical reflection and recommendations for optimising future interventions.

Ethical approval for the trial and the process evaluation was granted by the University of Western Sydney Human Research Ethics Committee, approval number H8970. Written informed consent is obtained from all study participants. Participant interviews are transcribed and de-identified. All data is kept confidential. The methods for monitoring and documenting intervention session delivery and obtaining participant feedback were piloted in a non-participant health centre within a government department before the trial began.

### Data collection

#### Domain 1: Implementation

Documentation of the intervention delivery is informed by the work of Bellg, Borrelli and colleagues who have developed frameworks for measuring intervention fidelity of individual health behaviour change treatments [22,25]. These frameworks were reviewed pragmatically for (a) applicability to the SPIRIT intervention change principles and (b) utility for our aims. Given the intervention’s complexity, the use of external content experts to deliver the program, and the likelihood of local tailoring, some items were adapted for improved fit, some were discarded (e.g., those that assessed participants’ comprehension and ability to perform skills), and a few were added (e.g., details of how interaction and reflective learning should be facilitated). Table 3 shows the final items (phrased as guiding questions) and the data collection strategies used for each.

**Table 3:** **Framework for documenting intervention implementation**

**Intervention implementation questions used to guide the documentation of intervention implementation**	**Data sources**				
	**SPIRIT team: engagement and design**	**SPIRIT team: implementation**	**Intervention session observations**	**Participant feedback**	**Participant interviews**
**Intervention design**					
1. What was the planned number & length of intervention sessions/activities, and their distribution and duration over time? Who were the targeted participants?	**✓**				
2. What theoretical model/theory-of-change were the strategies based on?	**✓**				
3. What *essential elements* were to be delivered in each component?	**✓**	**✓**			
**Intervention providers**					
4. What selection process was used to identify providers? What were the credentials of providers?	**✓**				
5. What training, guidance or information did providers receive (what was the content and format, and were there any changes over time?)?	**✓**				
**Recruitment**					
6. How were agencies recruited as participants?	**✓**				
7. What was the nature of the relationship between these agencies and the researchers/institutions involved in the trial?*	**✓**				**✓**
**Tailoring**					
8. Which intervention components did agency leaders select?		**✓**	**✓**		
9. What reasons did they give for that selection (what goals were they targeting)?		**✓**	**✓**		
10. What, if any, other goals and strategies were nominated by the agency leaders for supporting research use?		**✓**	**✓**		**✓**
**Intervention delivery**					
11. What method was used to specify and direct implementation?		**✓**	**✓**		
12. How long was each session?			**✓**		
13. What was the type, number and distribution of intervention sessions/activities?			**✓**		
14. To what extent were the *essential elements* delivered? How were they monitored/measured?			**✓**		
15. Were there any planned changes made to R4P while it was in progress? Why?		**✓**	**✓**	**✓**	
16. Were there any unplanned changes? What happened?		**✓**	**✓**		
**Context**					
17. What was the culture and overarching context of the participating agencies at the start of the intervention?*			**✓**		**✓**
18. Were there any changes/initiatives during the intervention that may have affected responses to the intervention?*			**✓**		**✓**
19. What were the immediate contextual conditions around intervention sessions?		**✓**	**✓**		
**Participation**					
20. Who was invited to participate? (numbers and professional roles)?		**✓**	**✓**		
21. How many potential participants attended sessions? Who were they?			**✓**		
22. What proportion of targeted participants attended (approximately)?			**✓**		
23. Did key people (agency leaders or topic specialists) attend?			**✓**		
**Responses to intervention sessions**					
24. How did people participate in intervention sessions?			**✓**	**✓**	**✓**
25. How satisfied were participants with sessions and SPIRIT overall?*			**✓**	**✓**	**✓**
26. Did participants identify or anticipate any changes in using research in response to intervention sessions/activities?*			**✓**	**✓**	**✓**
**Intervention improvements**	Analysis of data above				
27. What improvements to the intervention design and/or implementation are suggested by this data?*					
28. What lessons might be relevant to other interventions and settings?*					

*These items overlap with domains 2 &/or 3. They are included here because domain 1 data collection covers aspects of this information.

As shown in Table 2, four types of data are collected under Domain 1:

1. Updates from SPIRIT intervention staff: The evaluation officer meets regularly with SPIRIT staff who are working with agencies and external providers. Updates cover on-going developments regarding the selection of intervention components and tailored content, including what choices are made and why; and variations in processes used to recruit and brief providers. The evaluation officer attends weekly SPIRIT staff meetings and so is able to monitor operational processes, including why revisions are made to implementation plans.
2. Structured observation for fidelity assessment: Intervention delivery checklists are used to document core information about each session (dates, duration, etc.) and the extent to which each component’s essential elements are delivered. Checklists are scored quantitatively where possible and completed by the evaluation officer in each session using direct observation backed by digital audio recording. A scoring guide defines criteria for each essential element. A list of the essential elements and the participant feedback form for each session are sent to the session providers a week before delivery. See Additional file 2 for an example of the delivery checklist used for the SPIRIT Leadership Program session ‘Supporting organisational use of evidence.’
3. Email information: The SPIRIT trial coordinator keeps records of the content and dates of emails sent by SPIRIT staff (invitations to participate in sessions and in outcome measurement surveys) and by agency staff (CEO endorsements and invitations as above).
4. Knowledge brokering records: We use existing monitoring and evaluation processes for the brokered intervention component (component 4 in Table 1). These comprise standardised quality assurance interviews with the person at the agency commissioning the product, and the lead of the research team that develops the product. Interviews take place six months after the completion of the work and ask for reflections on the brokering process (satisfaction, efficiency), level of contact between the agency and the research team, and the utility of the final product [48].

#### Domain 2: Participation and response

Six types of data are collected to meet the Domain 2 objectives:

1. Pre-session sign-in and consent: Participants are asked to sign in at the beginning of sessions, state their job title/position and give or decline consent for process evaluation data collection (digital recording and note-taking). Information about professional roles allows us to document different types of reach and participation in each agency.
2. Semi-structured and structured observation: The evaluation officer observes and digitally records intervention sessions. The delivery checklist (described above) is used to collate structured information about participation and responses to SPIRIT. Descriptive field notes are taken to supply supporting data (e.g., examples of body language or interactions that illustrate the quality of participation) and to record information the checklist does not cover, such as how participants appear to interact with session contents, providers, and with each other; plus any contributions that might help answer our research questions. Notes are marked to indicate potentially valuable comments that should be verified using the audio recording. Immediately after each session, these notes are entered into a semi-structured session memo template in order to synthesise key aspects of the data and link it to other sources, and to explore hypotheses that will inform further data collection.
3. Self-reported evaluation feedback: Participants are asked to complete anonymous feedback forms immediately after intervention sessions. Session-specific questions derive from the SPIRIT change principles. Additional file 3 provides an example of the feedback form used for the Leadership Program session ‘Supporting organisational use of evidence.’
4. Informal conversations after sessions: When circumstances allow, the evaluation officer engages in conversation with participants about their views of the session, and wider implications of the topic/contents for their work. Providers are asked about their assessment of the session. Field notes are written immediately afterwards.
5. Interviews: Purposively sampled participants from each agency are interviewed at two points: early in their agency’s intervention period, and after the intervention has concluded. Interviewees include:
  - Each agency’s Liaison Person (the member of staff who has been nominated to support the administration and promotion of SPIRIT in their agency).
  - Up to six staff identified from their responses to an online survey conducted as part of the outcome measures. They are selected for the range of variation (the highest and lowest scores) in three Domains of this tool: valuing research, confidence in using research, and research use behaviours. Interviewees are not informed of their individual survey scores.
  - Further staff identified by the evaluation officer during observation, or by other interviewees, as being highly informative regarding research use in their agency.

The first round of interviews focuses on agency culture and context (see section below). The second round focuses on how the interviewee, their team and the wider organisation perceived and responded to the intervention and other aspects of SPIRIT such as the outcome measures and the process evaluation. A flow chart of open-ended questions and prompts derived from the SPIRIT program logic model is used to explore interviewees’ views and accounts of how the intervention may have influenced their, and their organisation’s, capacity to use research. A copy of the interview schedule for general participants (i.e., not the Liaison Person or CEO) is available in Additional file 4. All participant interviews are digitally recorded and professionally transcribed. An unstructured memo is written directly after each interview to capture initial analytic thinking and hypotheses. Memos are further developed when the transcriptions are read, corrected and de-identified.

6. Interviews, meetings and informal conversations: Throughout the trial, information is collected from the people implementing SPIRIT about participation and responses to the intervention, the outcome measures, and the process evaluation. Ad hoc conversations address issues such as how participants are responding to requests to complete outcome measures, and feedback from Liaison People about the administrative tasks they are engaged in. SPIRIT staff were interviewed after agency visits during the pre-intervention agency engagement phase, and continue to be interviewed after they provide mid-intervention feedback (these sessions are not attended by the evaluation officer). Questions focus on agency attitudes to SPIRIT and any factors that might affect engagement and outcomes.

#### Domain 3: Context

We conceptualised context as incorporating the social, structural and political environment of each participating organisation, but focus on six dimensions that we identified from the bodies of literature described earlier:

(i) work practices and culture;
(ii) agenda-setting and work prioritisation;
(iii) leadership styles and how leaders are perceived;
(iv) how different kinds of information, including research, is accessed, used and valued by individuals and the broader organisation;
(v) barriers and enablers to using research;
(vi) any other contextual factors that might affect outcomes.

Four types of data are collected under this Domain:

1. Structured observation: The delivery checklist and supplementary field notes are used to collate core information about the context of sessions (site, facilities, etc.).
2. Semi-structured observation: During intervention sessions, the evaluation officer takes extensive field notes in relation to the dimensions described above. This information is collated using the same methods as for the participation information (described above in Domain 2, data type 2: Semi-structured and structured observation).
3. Interviews: These take place with purposively sampled participants in the early phase of the intervention and focus on capturing information within the six dimensions outlined above. A copy of the interview schedule for general participants is available in Additional file 5. The CEO, or equivalent, for each agency will be invited to participate in an interview after the final round of outcome measures is complete. This interview will explore why they participated in the trial, what else was going on in and around the organisation that might have affected how staff engage with research, and how change does or does not occur in that organisation.
4. Interviews, meetings and informal conversations: The interviews, study management meetings, and informal conversations with SPIRIT staff described under Domain 2 also address contextual issues. This includes feedback from agencies about changes in funding, staff and governance; and how they are being affected by developments in external agencies, politics and the media.

Semi-structured running memos are maintained for each of the six organisations that capture information about participation, responses and contextual changes. They include additional information that is collected opportunistically from a variety of sources including ad hoc conversations with agency staff at non-SPIRIT forums (e.g., conferences) and electronic media such as Twitter and government websites. A cross-agency memo is maintained to capture overarching issues and themes, including emerging themes that require more investigation in the field.

### Program improvement

Interview and observational data are collected across each Domain to inform program improvement recommendations for future studies. For Domain 1 (implementation), we focus on how delivery might be improved. This includes fidelity considerations (the congruence between intended and actual delivery) and factors that are not specified in the implementation plan, such as day-to-day communication strategies and creative variations introduced by providers. In Domain 2 (participation and response), we ask how the intervention content, structure and techniques might have better met the needs of the targeted personnel in each agency and effected change more successfully. In Domain 3 (context), we focus on how each intervention setting may have influenced proximal outcomes (including participation) and distal outcomes as measured in the trial, and how the design and delivery of the intervention could have been more appropriate for and responsive to agency culture and context.

### Data management and analysis

#### Domain 1 (implementation)

Data from the delivery checklists and participant feedback forms is entered into a database that contains fields for each item by session and by agency. This provides a comparative overview of delivery fidelity, why intentional and unintentional changes are made, participants’ evaluative feedback, and other critical information about intervention sites, program delivery and participation. See Additional file 6 for an example of the spreadsheet used to collate data about a Leadership Program session. Analysis will focus on variation between agencies in how the intervention was implemented, particularly differences in the proportion of essential elements delivered at each site and differences in participant feedback, and any association between the two.

#### Domains 2 (participation and response) and 3 (context)

All other data (early- and post-intervention interview transcripts, and session, agency and interview memos) are managed using Framework Analysis [49,50]. Framework allows large amounts of diverse data to be analysed systematically, it is more transparent than most qualitative data analysis methods, it simplifies and supports comparative case analysis, and it enables us to review in-progress analysis as a team [49,50]. All transcripts are uploaded to NVivo 10 [51] and synthesised in matrices. We use three matrices: one for Domain 2 participation and response data; one for Domain 3 context data; and a third that collates data about participants’ research and information utilisation. Data are organised both by case (individuals clustered by agency) and by category. Categories were developed by the process evaluation team reviewing preliminary interview data and memos in relation to the process evaluation questions and the SPIRIT program logic model, and multiple coding interview transcripts to test and revise the categories. Categories include the range of intervention implementation strategies and the research engagement actions identified in the SPIRIT program logic model. Additional file 7 lists the categories used for Domain 2 (participation and response) and Domain 3 (context) framework matrices. Limited qualitative information from two outcome measures is included; the person conducting and coding the outcome measures interviews collates de-identified data from transcripts in relation to the six dimensions used to guide observations (described above) and provides it in a form that allows it to be integrated into the process evaluation framework matrices for analysis. Completed matrices thereby synthesise our varied data within broad categories, in preparation for more interpretive analysis [49]. Memos from interviews and intervention sessions, agency memos and the overarching cross-agency memo are re-read and coded in NVivo using Domain 2 and 3 framework categories.

All data related to each category is clustered and reviewed inductively, identifying key themes from close reading of the data — across all sessions and participants — to identify and distil the variation of views, experiences and behaviours within each agency. This work includes the development of schematic case studies for each agency. Interpretive memos are written that refine the theme, linking it to corroborating data sources and, when appropriate, linking it to other categories or themes in NVivo or in the implementation fidelity database so that each theme is supported by the broadest range of evidence. This data is reviewed in relation to the interactions between delivery, participation and context, and will also be reviewed in relation to outcomes when they are known. Analysis is on-going and guides continuing data collection. Early themes are revisited in the light of subsequent analytical changes [49,50]. Themes are explored within and across cases and will be reviewed in relation to the outcomes. The small process evaluation team, who co-designed the process evaluation and monitor its implementation, review distilled data and discuss interpretations.

## Trial status

SPIRIT is currently being implemented. The trial will conclude in April 2015.

## Discussion

In this paper, we describe the design of a process evaluation embedded within a trial of a complex intervention designed to build individual and organisational capacity to use research in policy and program development. We report the methods of the process evaluation and discuss how they are functioning during implementation. In doing so, we contribute to the literature in three ways: (i) we provide a worked example of how to embed process evaluation in the design and evaluation of a complex intervention (these are rare in the literature [15]); (ii) we illustrate an approach to tackling the challenges of complexity in the intervention and its implementation settings; and (iii) we provide, to our knowledge, the only detailed example of the methods for a process evaluation of an intervention conducted as part of a randomised trial in policy organisations.

### Strengths and weaknesses

As an integral part of the SPIRIT trial, the process evaluation is well-resourced, detailed and has good access to a range of rich data sources. Early trialling and consultation with policy and program colleagues helped us identify methods that would be appropriate, feasible and effective. For example, they advised us that focus groups would have low attendance and that interviews would obtain franker responses. Also, that our original plan to use ethnographic methods to study day-to-day work practices would be regarded as unacceptably intrusive, particularly in the context of an evaluation. To date, our methods appear to be appropriate for this trial. They are sufficiently flexible to gather data responsively and, with minor exceptions, there is no indication that the process evaluation has impacted participants’ comfort or willingness to express themselves frankly in intervention sessions. Participants have given consent for intervention sessions to be observed and recorded, and the majority have completed anonymous feedback forms with no negative comments about the process evaluation. External presenters have understood the purpose of fidelity monitoring and have not appeared to be affected by it. Observations of these sessions (particularly those that are highly interactive) provide access to nuanced information about norms, values, processes, priorities and constraints that is helping us develop rich case studies of each agency’s organisational context. The two phases of face-to-face interviews (early interviews focus on organisational culture and work processes, while post-intervention interviews focus on impact) are providing valuable insights about the relationships between the intervention, participation and context. Discussion with the multidisciplinary process evaluation team about emerging themes and interpretations strengthens the trustworthiness of findings.

However, we note some weaknesses. The role of the evaluation officer may inhibit full and frank feedback. Participants are aware that she works within the study team that includes the researchers responsible for designing and implementing the intervention, and some seem to assume that she is involved in decisions about design and implementation. This may affect the openness with which they talk about the trial. Also, the evaluation officer is a researcher asking participants about an intervention designed to increase how they value and use research. It is likely that she is not perceived as disinterested, and this may result in social desirability bias in interviews. The process evaluation itself may add to the burden of participation, and people may find it hard to raise this. To date, these factors do not appear to have had significant effects. As in previous interview-based studies with policymakers [52], respondents have been generous with their time in interviews and our impression is that most have spoken openly, but people with concerns may have been deterred from participating in interviews in the first place.

Lastly, comprehensive evaluation of the action framework is outside the scope of process evaluation, so our contribution to the evolution of the SPIRIT Action Framework will be limited. We should be able to comment on applicability of the Framework within the parameters of this trial, and to flesh out some of the nuances in the relationships between its component parts. But more targeted and responsive data collection and analysis is needed to generate hypotheses that can inform further iterations of the Framework. As in other investigations of policy processes to date, our methods do not fully access the central phenomenon of policy decision-making and the role (current and potential) that research plays in it.

## Conclusion

This paper presents a detailed protocol for the process evaluation of a unique complex intervention in health policy and program agencies. A key feature of the design is the development of flexible and pragmatic methods to capture data across three Domains: 1) How the intervention was delivered; 2) How people in each agency participated in and responded to the intervention; and 3) Contextual factors that may affect how the intervention was delivered and received. We provide examples of tools used. The data will be used to develop an understanding of how and why the intervention had the effects it did (or did not) in each setting, and to draw out implications for improving future interventions. In order to conduct a process evaluation in busy policy agencies, we had to develop methods that optimised data collection but minimised discomfort and burden for participants. We discuss some strengths and weakness associated with these methods, informed by reflections on the early implementation of the process evaluation. Given that we found little concrete guidance in the literature to help us develop these methods and tools, our account may provide a useful reference for others developing process evaluations for trials of complex interventions.

## Additional files

Additional file 1
**Explanation of terms used in this article.**
Additional file 2
**Example of a delivery checklist.**
Additional file 3
**Example of a feedback form.**
Additional file 4
**Post-intervention process evaluation interviews.**
Additional file 5
**Early process evaluation interviews.**
Additional file 6
**Spreadsheet for data management of Leadership program session: ‘Supporting organisational use of evidence’.**
Additional file 7
**Categories used for Domain 2 and Domain 3 Framework matrices.**
