# Supplementary material for: Protocol for the process evaluation of a complex intervention designed to increase the use of research in health policy and program organisations (the SPIRIT study)
Source: Implement Sci. 2014 Sep 27;9:113. doi: 10.1186/s13012-014-0113-0 (PMC4218994; doi:10.1186/s13012-014-0113-0)
Supplement: Additional file 1 — Explanation of terms used in this article. [file 13012_2014_113_MOESM1_ESM.docx]

## Additional file 1: Explanation of terms used in this article

| Term | Explanation |
| --- | --- |
| SPIRIT | *‘Supporting Policy in health with Research: an Intervention Trial’* is a study testing the effectiveness of an intervention on the use of research in health policy and program development. It comprises: an intervention trial using a stepped wedge design, outcome measures, and a process evaluation. |
| Intervention | The SPIRIT intervention is a 12 month multi-component intervention implemented in six health policy and program agencies in Sydney, Australia. Aspects of the intervention are tailored in response to each agency’s self-identified priorities and interests. |
| Intervention components | The intervention has 6 main components: 1. Audit, feedback and goal setting; 2. Leadership Program; 3. Agency Support; 4. Opportunity to test systems for accessing research and reviews; 5. Research exchanges; and 6. Staff symposia. Most components have several subcomponent delivered over the course of one year. See Table 1 for details. |
| Intervention sessions | *Sessions* refer to subcomponents of the intervention that are delivered face-to-face in a workshop format, making them observable, contextually responsive and allowing immediate participant feedback. As shown in Table 1, all components except no. 3 include one or more sessions. |
| Change principles | These were developed by SPIRIT investigators to provide overarching guidance for the design of the intervention and to identify goals for each subcomponent. The change principles that underpin each intervention component are shown in Table 1. |
| Fidelity | The extent to which the intervention is delivered as planned in each agency. |
| Essential elements | Aspects of the intervention which are hypothesised to be critical to the effectiveness of each component and the intervention overall, and must therefore be delivered. Additional file 3 shows the essential elements of a Leadership Program session: *Supporting organisational use of evidence*. |
| Participating agencies | Participating agencies are centres in government departments or statutory agencies that focus on health policy or program development and implementation. They were selected from a larger sample frame. All invited agencies agreed to participate and were randomly assigned to an intervention start point. |
| Liaison Person | Each participating agency nominated a member of staff to act as an intermediary and facilitator for the SPIRIT intervention and outcome measures in their agency. These Liaison People received an in-depth briefing, detailed background information and instructions, and are in regular contact with SPIRIT staff. |
| SPIRIT staff | SPIRIT staff are referred to in two groups: 1. those who were involved in engaging agencies and designing the intervention, and 2. those who are involved in implementing the intervention and outcome measures, including working with agencies to tailor the intervention to their needs. |
| SPIRIT providers | Leading researchers and high profile policymakers sought for their content specialism, impressive credentials and presentation/engagement skills. Providers are briefed about session goals, essential elements, participant feedback form contents and, where appropriate, the questions and concerns that each agency would like addressed, including any relevant case examples. |
