# Supplementary figures and images for: Protocol for the process evaluation of a complex intervention designed to increase the use of research in health policy and program organisations (the SPIRIT study)
Source: Implement Sci. 2014 Sep 27;9:113. doi: 10.1186/s13012-014-0113-0 (PMC4218994; doi:10.1186/s13012-014-0113-0)

## Additional file 2: SPIRIT Action Framework


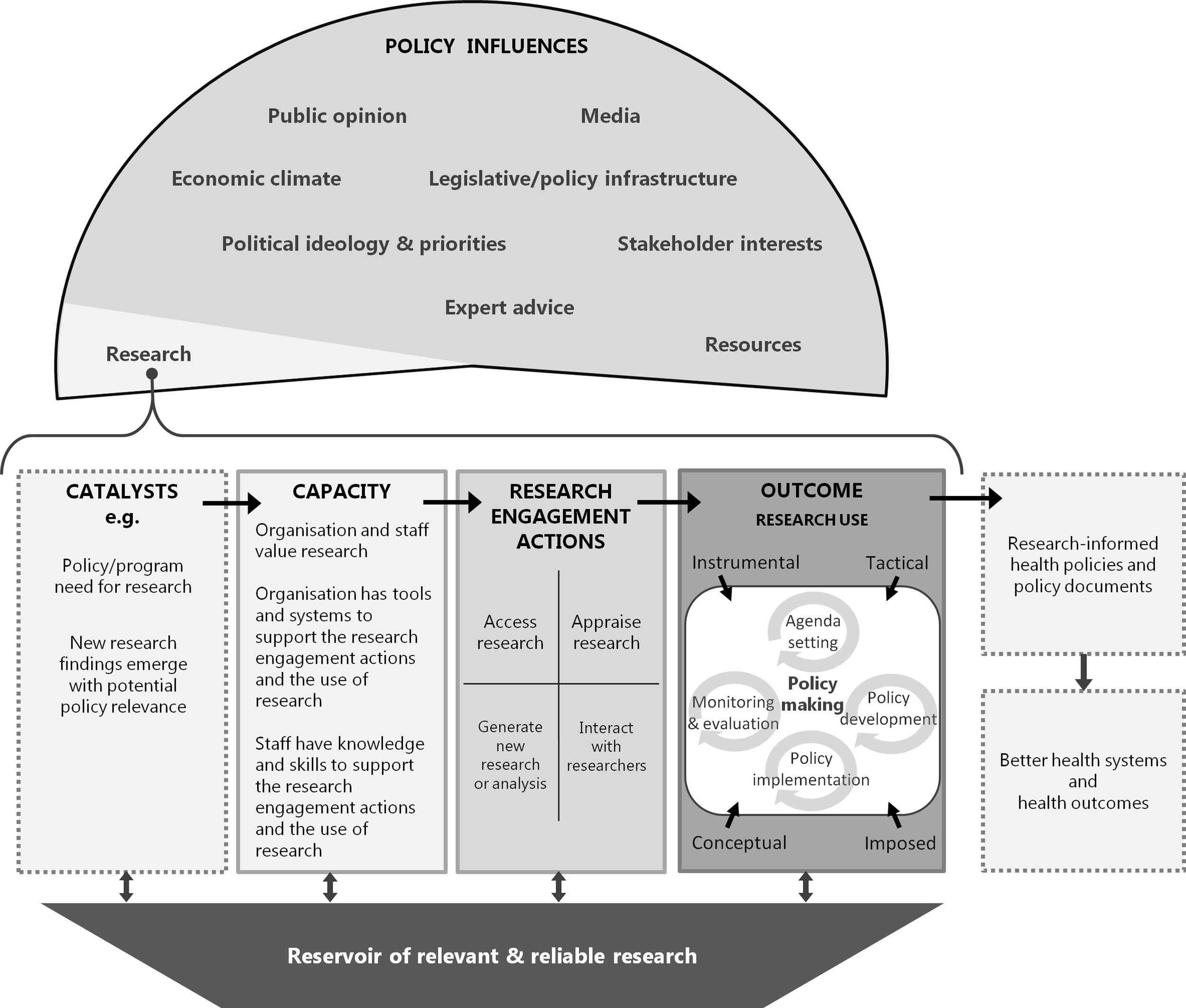

Supplement: Additional file 2 — Example of a delivery checklist. [file 13012_2014_113_MOESM2_ESM.docx]
