# Supplementary material for: Protocol for the process evaluation of a complex intervention designed to increase the use of research in health policy and program organisations (the SPIRIT study)
Source: Implement Sci. 2014 Sep 27;9:113. doi: 10.1186/s13012-014-0113-0 (PMC4218994; doi:10.1186/s13012-014-0113-0)
Supplement: Additional file 3 — Example of a feedback form. [file 13012_2014_113_MOESM3_ESM.docx]

##
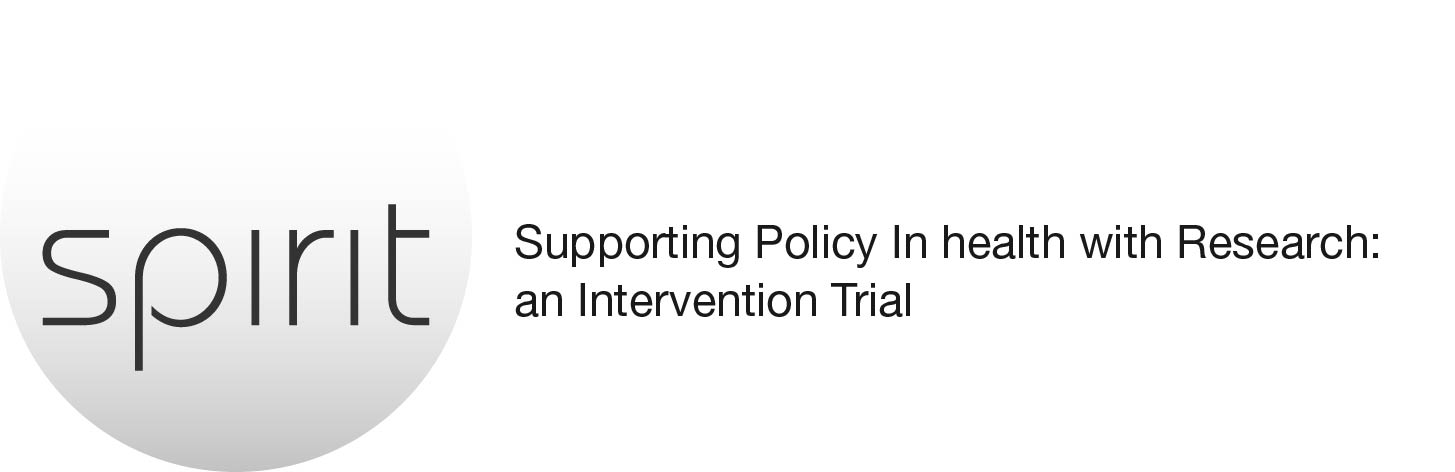
Additional file 3: Example of a delivery checklist

**Agency code**

**Delivery checklist**

**Session details**

| Component name | **Leadership program** | Session name | **Supporting organisational use of evidence** |
| --- | --- | --- | --- |
| Date of delivery |  | Name of session provider(s) |  |
| Mode of delivery |  | | |
| Attendance/consent sign in sheet used? |  | Any refusals of consent? |  |
| Audio recording? |  | Participant feedback forms? |  |
| Were there any handouts?  What were they? |  | | |

| Intended start time |  | Intended end time |  | Intended duration of session |  |
| --- | --- | --- | --- | --- | --- |
| Actual start time |  | Actual end time |  | Actual duration of session |  |

**Monitoring details**

| Date log sheet was completed |  | Name of evaluator |  |
| --- | --- | --- | --- |
| How monitoring was conducted | ❑ Direct observation by evaluator ❑ Audio recording of session  ❑ Direct review of module (e-Bulletin etc) ❑ Discussion with provider  ❑ Self-reported coding sheet completed by provider | | |

**Attendance**

| Number of participants – total  (may vary during session) |  |
| --- | --- |
| Participants’ roles – number of types of roles  (data available from sign-in/consent sheet) |  |
| Any other non-participant attendees (SPIRIT staff, uninvited agency staff, etc) |  |

**Essential element coding**

| **Essential elements** | **Code** |
| --- | --- |
| **Provider characteristics** | |
| Provider(s) had expertise and credentials appropriate to the session | Y / N |
| Provider(s)had experience in presenting to policy / program developers | Y / N |
| **Content and facilitation: Session plan** | |
| Core content in session plan was delivered [aggregate following codes]:   - Overview of international best practice in knowledge exchange - Potential barriers and facilitators for staff using research in policy/program work - Some strategies or tools for addressing barriers and/or encouraging their staff to use research | *Wholly \| Mostly \| About half \| Limited \| Not at all*  Y / N  Y / N  Y / N |
| **Content and facilitation: Component level** | |
| Content was delivered in an engaging manner* | *Extensive \| Moderate \| Limited \| Not at all* |
| The session content was relevant to the agency’s work* | *Extensive \| Moderate \| Limited \| Not at all* |
| Where specified in the session plan, provider identified or provided resources that supported or extended learning from the session | *Yes / Partially / No / N/A - not specified in plan* |
| Participants were encouraged to discuss 1> aspects of the topic | *Extensive \| Moderate \| Limited \| Not at all* |
| Participants were encouraged to discuss how learning from the session might be applied in their setting | *Extensive \| Moderate \| Limited \| Not at all* |
| Non-didactic teaching strategies were used; e.g. case studies, examples, quotes, demonstrations, pairs/small group discussion, practice activities | *Extensive \| Moderate \| Limited \| Not at all* |
| The value of using research in policy/program work was persuasively communicated | *Extensive \| Moderate \| Limited \| Not at all* |
| Provider showed respect for participants’ contributions and work | *Extensive \| Moderate \| Limited \| Not at all* |
| Provider showed sensitivity to the ‘real world’ of policy/program work | *Extensive \| Moderate \| Limited \| Not at all* |
| Opportunities to improve use of research were identified | *Extensive \| Moderate \| Limited \| Not at all* |
| **Participation** | |
| A leader (senior person in the agency e.g. member of the executive or equivalent) introduced the session | Y / N |
| Participants contributed to discussion | *All \| ~ 3/4 \| ~ 1/2 \| ~ 1/4 \| Few \| None* |
| Participants’ contributions included knowledge/examples from their own experience | *Extensive \| Moderate \| Limited \| Not at all* |
| Discussion included how info/learning from the session might be applied in their setting | *Extensive \| Moderate \| Limited \| Not at all* |
| Participants discussed some change goals | Y / N |

**Other notes**

| Any changes to the above plan? | Y  N | What and why? |  |
| --- | --- | --- | --- |

| Any pre or post forum activities? | Y  N | Did they occur as planned? |  |
| --- | --- | --- | --- |

* Refer to participant feedback form responses
