# Supplementary material for: Protocol for the process evaluation of a complex intervention designed to increase the use of research in health policy and program organisations (the SPIRIT study)
Source: Implement Sci. 2014 Sep 27;9:113. doi: 10.1186/s13012-014-0113-0 (PMC4218994; doi:10.1186/s13012-014-0113-0)
Supplement: Additional file 4 — Post-intervention process evaluation interviews. [file 13012_2014_113_MOESM4_ESM.docx]

##
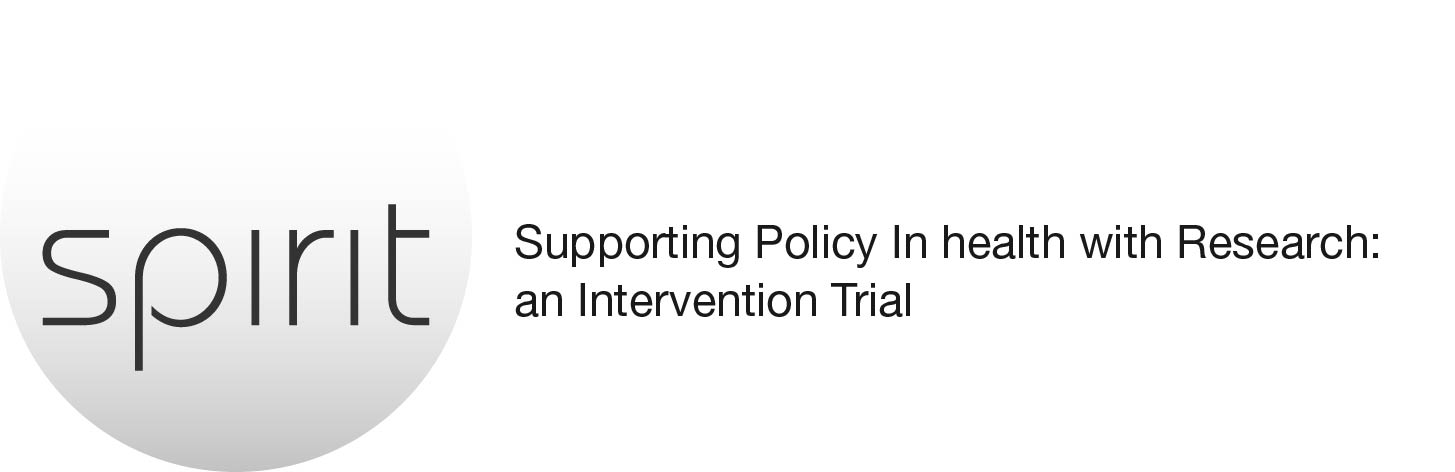
Additional file 4: Example of a feedback form

Feedback form

**Leaders’ forum: Supporting the Use of Evidence**

[Agency name and date of session]

**The forum:**  Please circle one option

|  | Was interesting and engaging | YES NO |
| --- | --- | --- |
|  | Had a presenter with appropriate knowledge and skills | YES NO |
|  | Provided useful information | YES NO |
| 1. 1 | Was relevant to my work | YES NO |
|  | Strengthened my view about the value of using research evidence | YES NO |

**Following the forum:**

|  | I have increased understanding of best practice in knowledge exchange | YES NO |
| --- | --- | --- |
|  | I can identify some potential barriers and facilitators for my staff in using research evidence in their work | YES NO |
|  | I can identify some strategies for supporting the use of research evidence in my organisation | YES NO |

**It is likely that:**

|  | I will use learning from this session | Likely Unlikely |
| --- | --- | --- |
|  | I will participate in future SPIRIT sessions | Likely Unlikely |
|  | I will encourage my staff to participate in SPIRIT | Likely Unlikely |
| 1. 1 | SPIRIT will benefit this agency | Likely Unlikely |

**What worked well?**

**What could be improved?**

**Any other comments?**
