# Supplementary material for: Protocol for the process evaluation of a complex intervention designed to increase the use of research in health policy and program organisations (the SPIRIT study)
Source: Implement Sci. 2014 Sep 27;9:113. doi: 10.1186/s13012-014-0113-0 (PMC4218994; doi:10.1186/s13012-014-0113-0)
Supplement: Additional file 5 — Early process evaluation interviews. [file 13012_2014_113_MOESM5_ESM.docx]

## Additional file 5: Post-intervention process evaluation interviews (general participants with some level of participation)

**Introduction**

Before we kick off I’d like to give you a bit of context for our conversation. We’re going to talk about the SPIRIT study. This study has three parts: 1. A program of workshops and resources that aim to improve how research is used in policy and program development, 2. Then there are three ways of measuring impacts of the program - an online survey and two types of interviews, these are being implemented every six months, and lastly, 3. A process evaluation which is exploring how the program worked (or didn’t work) in each organisation. The last one is my job and that’s what we’re doing today.

**1. I’d like to start by asking why you decided to participate in SPIRIT.**

**2. What were your general impressions of the program?**

**3. Which aspects were most positive?**

**4. Which were most negative?**

**5. Do you think SPIRIT had any impact on how research is used or perceived or supported in this organisation?**

Have you noticed any changes in :

- How **senior managers value or express value for R**?
- **Tools & systems** that support R use?
- Colleagues’ **confidence/values** towards using R?
- Colleagues **skills/knowledge** in using R?

No

Yes

Why do you think that was?

Can you describe the impact?

What would have needed to be different for it to have had an impact?

What was it about the program that enabled it to have an impact?

- Strategies/essential elements?
- Role of leaders?

What improvements would have helped re:

- Program content
- Program goals
- Delivery strategies
- Role of leadership

**6. Did the program have any effect on you?**

Do you think there have been any changes in:

- The **way you think or talk about the value of R** in your work?
- Your **knowledge, skills, or confidence** in using R?

No

Yes

Why do you think that was?

Can you describe the effect?

- Strategies/essential elements?
- Role of leaders?
- Role of colleagues/team?

What would have needed to be different for it to have had an impact?

What was it that made the difference?

1. **Were you aware of any changes in your views or attitudes towards the program over its duration?**
2. **How do you think your colleagues viewed SPIRIT? What differences might there be between your views and theirs?**
3. ***[If not already addressed...]* One of the aims of SPIRIT was to engage well with the people the program was offered to. To what extent did SPIRIT manage to engage staff here? What could have been done better?**

1. ***[If not already addressed...]* It’s probably safe to say that SPIRIT didn’t manage to engage everyone who was eligible to participate. Without naming anyone, can you think of any colleagues who, as far as you know, chose not to participate or resisted it? I appreciate that this may be speculative, but can you hazard any explanation for this?**
2. **The people who took on the role of facilitating SPIRIT in each organisation were quite diverse. In your organisation X acted in that role. Do you think her/his position here or the way she/he approached the tasks involved in facilitating SPIRIT might have affected how people engaged with it?**
3. **Assuming there’s always room for improvement, what advice would you give the people who designed and implemented the program about how to make it effective in agencies like this one?**
4. **I’d like you to erase SPIRIT from your mind now and imagine that *you* have been given the task of increasing the use of research in this organisation’s work. If there were no financial, structural or political restrictions, what would you do?**
5. **Imagining the same idealised world with no financial, structural or political restrictions , but in this scenario you have been given the task of improving the quality of policies/programs produced by this organisation in whatever way you see fit. What would you do?**
6. **Lastly, given that I’m trying to figure out how and why aspects of the program worked (or didn’t work) in different contexts, is there anything else it would be helpful for me to know?**
