# Supplementary material for: Protocol for the process evaluation of a complex intervention designed to increase the use of research in health policy and program organisations (the SPIRIT study)
Source: Implement Sci. 2014 Sep 27;9:113. doi: 10.1186/s13012-014-0113-0 (PMC4218994; doi:10.1186/s13012-014-0113-0)
Supplement: Additional file 6 — Spreadsheet for data management of Leadership program session: ‘Supporting organisational use of evidence’. [file 13012_2014_113_MOESM6_ESM.docx]

| Additional file 6: Early process evaluation interviews (general participants) | |
| --- | --- |
| **Introduction**  This interview is part of a larger evaluation of the SPIRIT study. My goal today is to understand as much as I can about how policy/program work is done here and how the culture of the organisation and the context you work in affect that. This will help us to assess how different aspects of the of the SPIRIT program work (or don’t work) in different contexts. | |
| **Work culture and context**   1. I’d like to get a sense of what you do day-to-day. Can you briefly describe the work you do, focusing on your main responsibilities? | |
| 1. I’m interested in how people’s work agendas are set. Thinking about the work that you and your immediate colleagues do, who or what has the most influence in determining what gets prioritised each day? | |
| 1. How would you describe the culture of this organisation? | |
| 1. What significant changes have taken place in your workplace over the past year or so?  - Have they / how have they impacted on you and your colleagues’? - How do you feel the organisation has coped / is coping with these changes? | |
| **The role of research and other information resources**   1. I’m interested in the sort of information that informs your work. I have some cards here with different types of information on them.* Can you tell me which of these you use to answer the sorts of questions you deal with in your program/policy work? | |
| 1. I’d like to know about the usefulness of these types of information. Can tell me how each of these are useful to you?    - What is this useful for? What affects how you use it? | |
| 1. How do you get hold of these types of information? | |
| 1. What makes it harder or easier to use these types of information? | |
| 1. How important is the trustworthiness (credibility, reliability) of these types of information?    - How do you judge whether they are trustworthy or not? | |
| 1. *[if research not selected: sum up overview of how and why selected information types are used, suggest why research was not included and ask if there are any other reasons]* | |
| 1. *[only if the interviewee uses some form of research...]Y*ou’ve described how you see the value of research in the mix of information you use. To what extent do you think your views are shared by your colleagues and by managers in this organisation?  - Where do you think these views come from? - Are there any examples? | |
| 1. The way that people use information in their work can be influenced by many things: their personal knowledge, skills and background, by their work role and responsibilities, and by the expectations and culture of the organisation they work in. What do you think particularly influences the way that you use [research and other types of] information in your work? | |
| 1. I’m interested in how organisations support staff to use research. What do you see as the strengths and weaknesses in how this organisation supports staff to use research?  - Are there any people here who particularly support or advocate for using research in policy/program work? | |
| **Research for Policy Program**  Before we finish, I’d like to talk about the SPIRIT program. You’ve probably only encountered one or two parts of this so far—you may have completed an online survey and possibly another interview, and you may have attended an information or feedback session. I’d like to ask you a couple of questions about your impression of the program at this early stage. | |
| 1. If you had 10 minutes with the team who designed and are implementing the program, what advice would you give them? | |
| 1. Based on your impression of the program so far, what is your prediction about how people will respond to it? Do you think it has the potential to change the way that research is used here? | |
| 1. Do you think it may have anything to offer you? | |
| 1. Given that my job is to evaluate how the SPITIT program works in real world contexts, is there anything else I should know that would help me understand how it may or may not work in this organisation? | |
| I would love to talk to you again in about 10 months’ time after the program is over. May I have your permission to contact you then to see if you would be willing to do a follow-up interview? | |

*** The types of information on the cards were:**

- Internal expertise / advice from colleagues
- Advice from researchers / academics
- Advice or reports from NGOs or professional bodies
- Advice or reports from private industry stakeholders
- Government reports
  - federal government
  - other states & territories
  - international jurisdictions
- Legislation
- Research commissioned by my organisation
- Research conducted by my organisation (independently or in partnership)
- Peer-reviewed research papers
- Research syntheses / systematic reviews
- Current guidelines or protocols
- Internal strategic plans / priority documents
- Internal progress reports and evaluations
- Feedback from patient / service user groups
- Consultation feedback
- Task forces / committees / advisory groups
- Data from authoritative websites
- News media articles or reports
- Public opinion polling data
- Social Media (e.g. Twitter, blogs)
