# Supplementary material for: Protocol for the process evaluation of a complex intervention designed to increase the use of research in health policy and program organisations (the SPIRIT study)
Source: Implement Sci. 2014 Sep 27;9:113. doi: 10.1186/s13012-014-0113-0 (PMC4218994; doi:10.1186/s13012-014-0113-0)
Supplement: Additional file 7 — Categories used for Domain 2 and Domain 3 Framework matrices. [file 13012_2014_113_MOESM7_ESM.docx]

| Additional file 7: Spreadsheet for data management of Leadership program session: ‘Supporting organisational use of evidence’  \| **Database fields for Leadership program session: Supporting organisational use of evidence** \| **Agencies** \| \| \| \| \| \| \| \| \| \| \| \| \| --- \| --- \| --- \| --- \| --- \| --- \| --- \| --- \| --- \| --- \| --- \| --- \| --- \| \| **1** \| \| **2** \| \| **3** \| \| **4** \| \| **5** \| \| **6** \| \| \| **Checklist items and other core session information** \| \| \| \| \| \| \| \| \| \| \| \| \| \| Sequence of delivery \|  \| \|  \| \|  \| \|  \| \|  \| \|  \| \| \| Session date \|  \| \|  \| \|  \| \|  \| \|  \| \|  \| \| \| Mode of delivery \|  \| \|  \| \|  \| \|  \| \|  \| \|  \| \| \| No. of attendees \|  \| \|  \| \|  \| \|  \| \|  \| \|  \| \| \| Number of attendees by role \|  \| \|  \| \|  \| \|  \| \|  \| \|  \| \| \| Proportion of targeted participants attending \|  \| \|  \| \|  \| \|  \| \|  \| \|  \| \| \| Non-participant attendees (SPIRIT) \|  \| \|  \| \|  \| \|  \| \|  \| \|  \| \| \| Non-participant attendees (other) \|  \| \|  \| \|  \| \|  \| \|  \| \|  \| \| \| Any notable non attendees \|  \| \|  \| \|  \| \|  \| \|  \| \|  \| \| \| Who was invited \|  \| \|  \| \|  \| \|  \| \|  \| \|  \| \| \| How were they invited \|  \| \|  \| \|  \| \|  \| \|  \| \|  \| \| \| What info did attendees have about session in advance \|  \| \|  \| \|  \| \|  \| \|  \| \|  \| \| \| Presenter(s) \|  \| \|  \| \|  \| \|  \| \|  \| \|  \| \| \| Presenter briefing and feedback \|  \| \|  \| \|  \| \|  \| \|  \| \|  \| \| \| Intended duration of session \|  \| \|  \| \|  \| \|  \| \|  \| \|  \| \| \| Actual duration \|  \| \|  \| \|  \| \|  \| \|  \| \|  \| \| \| Attendance/consent sign in sheet used \|  \| \|  \| \|  \| \|  \| \|  \| \|  \| \| \| Any refusals of consent \|  \| \|  \| \|  \| \|  \| \|  \| \|  \| \| \| Audio recorded \|  \| \|  \| \|  \| \|  \| \|  \| \|  \| \| \| Participant feedback forms \|  \| \|  \| \|  \| \|  \| \|  \| \|  \| \| \| Handouts \|  \| \|  \| \|  \| \|  \| \|  \| \|  \| \| \| How was process evaluation conducted \|  \| \|  \| \|  \| \|  \| \|  \| \|  \| \| \| By whom \|  \| \|  \| \|  \| \|  \| \|  \| \|  \| \| \| Session attended by that agency's SPIRIT Officer \|  \| \|  \| \|  \| \|  \| \|  \| \|  \| \| \| **Component-specific essential elements delivered in this session** \| \| \| \| \| \| \| \| \| \| \| \| \| \| Provider had expertise and credentials in the topic/field appropriate to the session \|  \| \|  \| \|  \| \|  \| \|  \| \|  \| \| \| Provider had experience in presenting to policy/program developers \|  \| \|  \| \|  \| \|  \| \|  \| \|  \| \| \| Providers were briefed by SPIRIT staff, including session goals and desired delivery style \|  \| \|  \| \|  \| \|  \| \|  \| \|  \| \| \| Providers were sent the fidelity codes and feedback form for their session a week before session \|  \| \|  \| \|  \| \|  \| \|  \| \|  \| \| \| Core content outlined in session plan was delivered *[use feedback forms for final score]*:   1. Overview of international best practice in knowledge exchange 2. Potential barriers and facilitators for staff using research 3. Some strategies or tools for addressing barriers and/or encouraging their staff to use research \|  \| \|  \| \|  \| \|  \| \|  \| \|  \| \| \| Where specified in the session plan, provider identified or provided resources that supported or extended \|  \| \|  \| \|  \| \|  \| \|  \| \|  \| \| \| The session content was relevant to the agency’s work \|  \| \|  \| \|  \| \|  \| \|  \| \|  \| \| \| Content was delivered in an engaging manner \|  \| \|  \| \|  \| \|  \| \|  \| \|  \| \| \| Participants encouraged to discuss one or more aspects of the topic \|  \| \|  \| \|  \| \|  \| \|  \| \|  \| \| \| Participants were encouraged to discuss how information/learning from the session might be applied in their setting \|  \| \|  \| \|  \| \|  \| \|  \| \|  \| \| \| Non-didactic teaching strategies were used \|  \| \|  \| \|  \| \|  \| \|  \| \|  \| \| \| The value of using research[1] in policy/program work was persuasively communicated \|  \| \|  \| \|  \| \|  \| \|  \| \|  \| \| \| Provider showed respect for participants’ contributions and work \|  \| \|  \| \|  \| \|  \| \|  \| \|  \| \| \| Provider demonstrated sensitivity to the ‘real world’ of policy/program work \|  \| \|  \| \|  \| \|  \| \|  \| \|  \| \| \| Opportunities to improve use of research were identified \|  \| \|  \| \|  \| \|  \| \|  \| \|  \| \| \| Session introduced by leader in agency \|  \| \|  \| \|  \| \|  \| \|  \| \|  \| \| \| Participants contributed to discussion \|  \| \|  \| \|  \| \|  \| \|  \| \|  \| \| \| Participant contributions included knowledge/examples from their own experience \|  \| \|  \| \|  \| \|  \| \|  \| \|  \| \| \| Discussion included how information/learning from the session might be applied in their setting \|  \| \|  \| \|  \| \|  \| \|  \| \|  \| \| \| Participants discussed some change goals \| \|  \| \|  \| \|  \| \|  \| \|  \| \|  \| \| **Descriptive notes** \| \| \| \| \| \| \| \| \| \| \| \| \| \| Any changes in implementation plan \| \|  \| \|  \| \|  \| \|  \| \|  \| \|  \| \| Style of presentation \| \|  \| \|  \| \|  \| \|  \| \|  \| \|  \| \| Role and presentation of SPIRIT Officer in session \| \|  \| \|  \| \|  \| \|  \| \|  \| \|  \| \| Role and presentation of Liaison Person in session \| \|  \| \|  \| \|  \| \|  \| \|  \| \|  \| \| Descriptive overview of participation \| \|  \| \|  \| \|  \| \|  \| \|  \| \|  \| \| Process evaluation conduct \| \|  \| \|  \| \|  \| \|  \| \|  \| \|  \| \| Implications for program improvements \| \|  \| \|  \| \|  \| \|  \| \|  \| \|  \| \| Impacts on engagement / outcomes - summary thoughts \| \|  \| \|  \| \|  \| \|  \| \|  \| \|  \| \| **Component-specific feedback forms items used for this session** \| \| \| \| \| \| \| \| \| \| \| \| \| \| The session… \| \| \| \| \| \| \| \| \| \| \| \| \| \| … Was interesting and engaging \| \|  \| \|  \| \|  \| \|  \| \|  \| \|  \| \| … Had a presenter with appropriate knowledge and skills \| \|  \| \|  \| \|  \| \|  \| \|  \| \|  \| \| … Provided useful information \| \|  \| \|  \| \|  \| \|  \| \|  \| \|  \| \| … Was relevant to my work \| \|  \| \|  \| \|  \| \|  \| \|  \| \|  \| \| … Strengthened my view about the value of using research evidence \| \|  \| \|  \| \|  \| \|  \| \|  \| \|  \| \| Following the session… \| \| \| \| \| \| \| \| \| \| \| \| \| \| … I have increased understanding of best practice in knowledge exchange \| \|  \| \|  \| \|  \| \|  \| \|  \| \|  \| \| … I can identify some potential barriers and facilitators for my staff in using research evidence in their work \| \|  \| \|  \| \|  \| \|  \| \|  \| \|  \| \| … I can identify some strategies for supporting the use of research evidence in my organisation \| \|  \| \|  \| \|  \| \|  \| \|  \| \|  \| \| It is likely I will use learning from this session \| \|  \| \|  \| \|  \| \|  \| \|  \| \|  \| \| It is likely I will participate in future sessions \| \|  \| \|  \| \|  \| \|  \| \|  \| \|  \| \| It is likely I will encourage my staff to participate in SPIRIT \| \|  \| \|  \| \|  \| \|  \| \|  \| \|  \| \| It is likely SPIRIT will benefit this agency \| \|  \| \|  \| \|  \| \|  \| \|  \| \|  \| \| What worked well? \| \|  \| \|  \| \|  \| \|  \| \|  \| \|  \| \| What could be improved? \| \|  \| \|  \| \|  \| \|  \| \|  \| \|  \| \| Other comments \| \|  \| \|  \| \|  \| \|  \| \|  \| \|  \| \| **Data management** \| \|  \| \|  \| \|  \| \|  \| \|  \| \|  \| \| Memo in NVivo \| \|  \| \|  \| \|  \| \|  \| \|  \| \|  \| \| Memo indexed \| \|  \| \|  \| \|  \| \|  \| \|  \| \|  \| |
| --- | --- | --- | --- | --- | --- | --- | --- | --- | --- | --- | --- | --- | --- | --- | --- | --- | --- | --- | --- | --- | --- | --- | --- | --- | --- | --- | --- | --- | --- | --- | --- | --- | --- | --- | --- | --- | --- | --- | --- | --- | --- | --- | --- | --- | --- | --- | --- | --- | --- | --- | --- | --- | --- | --- | --- | --- | --- | --- | --- | --- | --- | --- | --- | --- | --- | --- | --- | --- | --- | --- | --- | --- | --- | --- | --- | --- | --- | --- | --- | --- | --- | --- | --- | --- | --- | --- | --- | --- | --- | --- | --- | --- | --- | --- | --- | --- | --- | --- | --- | --- | --- | --- | --- | --- | --- | --- | --- | --- | --- | --- | --- | --- | --- | --- | --- | --- | --- | --- | --- | --- | --- | --- | --- | --- | --- | --- | --- | --- | --- | --- | --- | --- | --- | --- | --- | --- | --- | --- | --- | --- | --- | --- | --- | --- | --- | --- | --- | --- | --- | --- | --- | --- | --- | --- | --- | --- | --- | --- | --- | --- | --- | --- | --- | --- | --- | --- | --- | --- | --- | --- | --- | --- | --- | --- | --- | --- | --- | --- | --- | --- | --- | --- | --- | --- | --- | --- | --- | --- | --- | --- | --- | --- | --- | --- | --- | --- | --- | --- | --- | --- | --- | --- | --- | --- | --- | --- | --- | --- | --- | --- | --- | --- | --- | --- | --- | --- | --- | --- | --- | --- | --- | --- | --- | --- | --- | --- | --- | --- | --- | --- | --- | --- | --- | --- | --- | --- | --- | --- | --- | --- | --- | --- | --- | --- | --- | --- | --- | --- | --- | --- | --- | --- | --- | --- | --- | --- | --- | --- | --- | --- | --- | --- | --- | --- | --- | --- | --- | --- | --- | --- | --- | --- | --- | --- | --- | --- | --- | --- | --- | --- | --- | --- | --- | --- | --- | --- | --- | --- | --- | --- | --- | --- | --- | --- | --- | --- | --- | --- | --- | --- | --- | --- | --- | --- | --- | --- | --- | --- | --- | --- | --- | --- | --- | --- | --- | --- | --- | --- | --- | --- | --- | --- | --- | --- | --- | --- | --- | --- | --- | --- | --- | --- | --- | --- | --- | --- | --- | --- | --- | --- | --- | --- | --- | --- | --- | --- | --- | --- | --- | --- | --- | --- | --- | --- | --- | --- | --- | --- | --- | --- | --- | --- | --- | --- | --- | --- | --- | --- | --- | --- | --- | --- | --- | --- | --- | --- | --- | --- | --- | --- | --- | --- | --- | --- | --- | --- | --- | --- | --- | --- | --- | --- | --- | --- | --- | --- | --- | --- | --- | --- | --- | --- | --- | --- | --- | --- | --- | --- | --- | --- | --- | --- | --- | --- | --- | --- | --- | --- | --- | --- | --- | --- | --- | --- | --- | --- | --- | --- | --- | --- | --- | --- | --- | --- | --- | --- | --- | --- | --- | --- | --- | --- | --- | --- | --- | --- | --- | --- | --- | --- | --- | --- | --- | --- | --- | --- | --- | --- | --- | --- | --- | --- | --- | --- | --- | --- | --- | --- | --- | --- | --- | --- | --- | --- | --- | --- | --- | --- | --- | --- | --- | --- | --- | --- | --- | --- | --- | --- | --- | --- | --- | --- | --- | --- | --- | --- | --- | --- | --- | --- | --- | --- | --- | --- | --- | --- | --- | --- | --- | --- | --- | --- | --- | --- | --- | --- | --- | --- | --- | --- | --- | --- | --- | --- | --- | --- | --- | --- | --- | --- | --- | --- | --- | --- | --- | --- | --- | --- | --- | --- | --- | --- | --- | --- | --- | --- | --- | --- | --- | --- | --- | --- | --- | --- | --- | --- | --- | --- | --- | --- | --- | --- | --- | --- | --- | --- | --- | --- | --- | --- | --- | --- | --- | --- | --- | --- | --- | --- | --- | --- | --- | --- | --- | --- | --- | --- | --- | --- | --- | --- | --- | --- | --- | --- | --- | --- | --- | --- | --- | --- | --- | --- | --- | --- | --- | --- | --- | --- | --- | --- | --- | --- | --- | --- | --- | --- | --- | --- | --- | --- | --- | --- | --- | --- | --- | --- | --- | --- | --- | --- | --- | --- | --- | --- | --- | --- | --- | --- | --- | --- | --- | --- | --- | --- | --- | --- | --- | --- | --- | --- | --- | --- | --- | --- | --- | --- | --- | --- | --- | --- | --- | --- | --- | --- | --- | --- | --- | --- | --- | --- | --- | --- | --- | --- | --- | --- | --- | --- | --- | --- | --- | --- | --- | --- | --- | --- | --- | --- | --- | --- | --- | --- | --- | --- | --- | --- | --- | --- | --- | --- | --- | --- | --- | --- | --- | --- | --- | --- | --- | --- | --- | --- | --- | --- | --- | --- | --- | --- | --- | --- | --- | --- | --- | --- | --- | --- | --- | --- | --- | --- | --- | --- | --- | --- | --- | --- | --- | --- | --- | --- | --- | --- | --- | --- | --- | --- | --- | --- | --- | --- | --- | --- | --- | --- | --- | --- | --- | --- | --- | --- | --- | --- | --- | --- | --- | --- | --- | --- | --- | --- | --- | --- | --- | --- | --- | --- | --- | --- | --- | --- | --- | --- | --- | --- | --- | --- | --- | --- | --- | --- | --- | --- | --- | --- | --- | --- | --- | --- | --- | --- | --- | --- | --- | --- | --- | --- | --- | --- | --- | --- | --- | --- | --- | --- | --- | --- | --- | --- | --- | --- | --- | --- | --- | --- | --- | --- | --- | --- | --- | --- | --- | --- | --- | --- | --- | --- | --- | --- | --- | --- | --- | --- | --- | --- | --- | --- | --- | --- | --- | --- | --- | --- | --- | --- | --- | --- | --- | --- | --- | --- | --- | --- | --- | --- | --- | --- | --- | --- | --- | --- | --- | --- | --- | --- | --- | --- | --- | --- | --- | --- | --- | --- | --- | --- | --- | --- | --- | --- | --- | --- | --- | --- | --- | --- | --- | --- | --- | --- | --- | --- | --- | --- | --- | --- | --- | --- | --- | --- | --- | --- | --- | --- | --- | --- | --- | --- | --- | --- | --- | --- | --- | --- | --- | --- | --- | --- | --- | --- | --- | --- | --- | --- | --- | --- | --- | --- | --- | --- | --- | --- | --- | --- | --- | --- | --- | --- | --- | --- | --- | --- | --- | --- | --- | --- | --- | --- | --- | --- | --- | --- | --- | --- | --- | --- | --- | --- | --- | --- | --- | --- | --- | --- | --- | --- | --- | --- | --- | --- | --- | --- | --- | --- | --- | --- | --- | --- | --- | --- | --- | --- | --- | --- | --- | --- | --- | --- | --- | --- | --- | --- | --- | --- | --- | --- | --- | --- | --- | --- | --- | --- | --- | --- | --- |

Note: the actual spreadsheet has much wider columns for each agency due to the descriptive contents and collation of participant comments in the feedback forms.
